# The Wellness Quest: A health literacy and self‐advocacy tool developed by youth for youth mental health

**DOI:** 10.1111/hex.13214

**Published:** 2021-02-26

**Authors:** Asavari Syan, Janice Y. Y. Lam, Christal G. L. Huang, Maverick S. M. Smith, Karleigh Darnay, Lisa D. Hawke, Joanna Henderson

**Affiliations:** ^1^ Centre for Addiction and Mental Health Toronto ON Canada; ^2^ University of Toronto Toronto ON Canada

**Keywords:** mental health, self‐advocacy, service navigation, service user‐led, youth, youth engagement

## Abstract

**Background:**

Less than 20% of youth who experience mental health difficulties access and receive appropriate treatment. This is exacerbated by barriers such as stigma, confidentiality concerns and lack of mental health literacy. A youth team developed the Wellness Quest: a health literacy tool to enable help‐seeking youth to advocate for themselves.

**Objective:**

To evaluate the content, presentation and utility of the Wellness Quest tool among youth.

**Participants:**

Participants aged 14 to 26.

**Methods:**

A youth research team conducted five focus groups and one online survey to evaluate the Wellness Quest tool. Thematic analysis was used to analyse the qualitative data, and descriptive statistics were used to explore the survey results.

**Main results:**

Overall evaluations of the Wellness Quest were positive: participants felt it would be useful during their mental health help‐seeking journey. Participants expressed the need for information about services for specific populations, such as Indigenous, immigrants, refugees and 2SLGBTQ + youth. They expressed that the tool should be available in complementary online and print versions.

**Discussion:**

Improving mental health literacy may improve mental health by enabling youth and those who support them to recognize and respond to signs of distress and understanding where and how to get help. The Wellness Quest tool may equip youth with the knowledge to make informed decisions and advocate for their own mental health, thereby facilitating help‐seeking among youth.

**Patient or public contribution:**

Youth as service users led all stages of the project, from designing and conducting the study and analysing the data to writing the manuscript.

## 1 BACKGROUND

Half of all mental illnesses begin by the age of 14 and three‐quarters by mid‐20s.^1^ Mental health difficulties and illnesses are associated with negative effects on relationships, employment, physical health and general well‐being.^2^ Within Canada, an estimated 1.2 million children and youth are affected by mental health difficulties. However, <20% access and receive appropriate treatment.^3^

When faced with managing their mental health difficulties, youth are concerned about stigmatization and often prefer self‐reliance.^4^ They are also concerned about confidentiality, particularly as they are in a transitional stage of reducing their dependence on parents or caregivers.^5^ A preference for self‐reliance and poor mental health literacy are seen as important barriers to help‐seeking for young people.^6^, ^7^ Mental health literacy includes knowledge about the symptoms and effects of mental health difficulties, the types of help available, and how to access help. Strong mental health literacy in young people may lead to better outcomes by facilitating early help‐seeking.^8^

In addition to these barriers, navigating the mental health system can be described as a ‘maze’.^9^ People with mental health difficulties experience emotional and social strains, while also attempting to navigate their way through an often unfamiliar and fragmented mental health‐care system.^10^ Most young people accessing care for the first time find it difficult to navigate the mental health system with regard to how it is structured and the services that are offered,^5^, ^11^ thereby increasing the treatment gap. In order to address this issue, the input of youth is crucial to inform the design of interventions and resources that meet the needs of young people.^12^

Service user‐led research has been increasingly recognized as providing vital and authentic insight into mental health recovery.^13^ Engagement and collaboration with service users have enabled the exploration of under‐investigated aspects of the lived experience of mental health recovery, not only in directing the research, but also in making it relevant to their own contextual experience.^13^, ^14^ Benefits of service user‐led research include more control over research, independence, and focus on issues that are important for service users themselves.^15^ Partnerships with young people with lived and living expertise benefit both service development initiatives and research.^16^ These partnerships provide numerous reciprocal benefits, such as skill development and social engagement for the youth, as well as an increase in the feasibility, youth‐friendliness and ecological validity of the research.^17^ There are numerous examples of successful youth‐led health studies across multiple settings and locations.^18^, ^19^, ^20^ For example, in a study among street‐involved youth, participants reported that the service user‐led approach positively impacted the quality and quantity of data that were collected from other street‐involved youth participants.^20^In such studies, youth have made substantial contributions to creating health programmes and services that can better meet the needs of the youth.^21^

### 1.1 McCain Model of Youth Engagement

The McCain Model of Youth Engagement^16^ was developed by the Margaret and Wallace McCain Centre for Child, Youth and Family Mental Health at the Centre for Addiction and Mental Health (CAMH) in Toronto, Canada. Co‐created by youth and researchers, the model supports the creation of meaningful working relationships between youth and researchers, such that they work and make decisions together and learn from each other. Key attributes of the McCain Model are flexibility, mentorship, authentic decision making, and reciprocal learning. The McCain Centre has been successful in using this model to engage youth and young adults in health‐care research and advocacy initiatives.^16^ An important part of the Youth Engagement Initiative stemming from the McCain Model is the National Youth Action Council (NYAC). Created by the McCain Centre, NYAC is a group of over 120 young Canadians between the ages of 16 and 29 who work within the McCain Centre's Youth Engagement Initiative to create and implement youth‐led mental health projects.

### 1.2 The Wellness Quest project

Wellness Quest is a health literacy and self‐advocacy tool developed by NYAC that aims to help treatment‐seeking youth advocate for themselves in their mental health care. Developed by youth, for youth, the Wellness Quest project aims to move young people from the role of patient to partner in their mental health care. The Wellness Quest tool is composed of a checklist and a guide. The checklist helps young people identify the issues and services that are most important to them. It lists services, potential partners in treatment, and accessibility concerns, so that service providers and youth can work together to develop the best treatment plan for that individual. The guide provides an explanation of these issues and services in an engaging, easy‐to‐navigate format, with youth‐friendly language. The guide provides detailed information to help youth complete the checklist, so that they can use it as a tool for conversation with their service provider(s). The guide also suggests questions for young people to ask their service provider to ensure they are well informed. The checklist and guide work hand in hand to assist and educate young people seeking treatment, with the goal of self‐advocacy.

### 1.3 Objective

This youth‐led study evaluated the Wellness Quest tool through focus groups and a national survey to understand youth perspectives about the Wellness Quest tool's content, presentation, utility, and ability to promote self‐advocacy, to guide the next stage in tool development.

## 2 METHOD

To assess the Wellness Quest tool content, presentation and utility, and ability to promote self‐advocacy, a team of eight young people from NYAC took the tool into their own communities to be evaluated in focus groups of young people aged 16 to 26. This age range is within the definition of youth suggested by Statistics Canada, that is 15‐29.^22^ A national online survey was also conducted by young people from the McCain Centre Youth Engagement Initiative. The study was approved by the CAMH Research Ethics Board.

### 2.1 Participants

Five focus group consultations were conducted in five provinces across Canada in locations ranging from large urban centres to a smaller town ‐ Calgary (Alberta), Toronto (Ontario), Winnipeg (Manitoba), Saskatoon (Saskatchewan), and Grand Falls‐Windsor (Newfoundland) ‐ with a total of 52 participants. For a city to be included, they needed to have at least three unique mental health services available to young people, to ensure that participants could reflect on the tool in the context of local service availability.

An anonymous survey was available nationwide. Twenty‐five participants provided data on at least the first section on the content of the scale and were included in the analyses.

To be eligible, the young person had to be between the ages of 16 and 26 and reside in Canada. Due to a mistaken protocol deviation, one youth under the age of 16 was recruited into the study, that is a 14 year old. The Research Ethics Board was informed of this deviation and approved the inclusion of their data in the study. Recruitment for both the focus groups and online survey occurred through posters shared via Facebook, Twitter and email, to pre‐existing youth networks as identified by our Youth Engagement team. Additionally, posters were physically visible in the participating community centres in which focus groups were conducted and youth leads shared the posters within their networks.

### 2.2 Youth Engagement

As this project was youth‐led, the research team was comprised solely of youth, with experienced researchers who acted as consultants. Governed by the McCain Model, multiple levels of Youth Engagement were available, including high engagement for a small number of youth (youth leads) and more limited, short‐term engagement for a larger number of youth (youth research assistants, youth RAs) (see Table 1). Over the course of the project, there were four youth leads who led the different phases of the project: tool development, data collection, data analysis and manuscript writing. These were hired CAMH research staff who underwent necessary research training. Additionally, interested members of NYAC were recruited as youth RAs, who received honoraria for their contributions. Youth leads and RAs self‐identified as having experience with mental health difficulties, which was required to ensure that they had personal knowledge of challenges associated with mental health, were aware of the mental health system, and could use these experiences to inform their work.

**TABLE 1:** Description of Youth Engagement during all three phases of the project

Phase of project	Role of youth lead staff	Role of youth RAs	Number of youth engaged
Tool development	Initiation of project and objectives, development of tool	Supporting youth staff with initiation of project and objectives, development of tool	18
Data collection	Submit REB application, present project at conferences, onboard and train youth RAs, support youth RAs during focus groups	Locating setting for focus groups, facilitating focus groups	8
Data analysis and manuscript writing	Data analysis and manuscript writing	Assisting youth staff and providing feedback on manuscript and data analysis	6
		Total number of youth engaged^a^	29

^a^ Some youth were engaged in multiple phases.

Youth RAs from each community facilitated the focus group consultations to ensure that all facilitators had prior knowledge of the local context and resources. Training was provided to all youth RAs, which included the Tri‐Council Policy Statement: Ethical Conduct for Research Involving Humans (TCPS‐2^23^) ethics training, as well as a remotely delivered ethics training co‐led by youth and researchers, attendance of two focus group training sessions, and regular phone and email check‐ins with CAMH youth leads.

### 2.3 Setting

Host agencies were identified in each community by the youth RA, with support from the youth lead. Youth RAs were asked to identify community organizations that were both accessible and offered a youth‐friendly environment. The host organizations provided the physical space and clinical staff support.

### 2.4 Procedure

#### 2.4.1 Focus groups

The youth RAs provided potential participants with project information and a draft copy of the Wellness Quest tool electronically at least one week prior to the consultation to allow participants time to read the tool. On the day of the consultation, participants were presented an overview of the tool and study, then signed an informed consent form and completed a demographics questionnaire. The focus groups were facilitated by youth RAs and supported by a CAMH youth lead via phone or in person. Clinical support was available on site.

Each two‐hour focus group consisted of a moderated discussion of the Wellness Quest tool using a semi‐structured interview guide. After ‘ice breaker’ questions, the conversation focused on the content, presentation and utility of the tool specific to the community and province. Participants received honoraria for their participation. Focus groups were audio‐recorded.

#### 2.4.2 Online survey

An anonymous survey was hosted online through the REDCap^24^ data capture system and included multiple choice and short answer questions about the Wellness Quest tool, taking approximately an hour to complete.

The first page of the survey provided an informed consent form, followed by a short demographics questionnaire. The survey questions focused on the content, presentation and utility of the tool. At the end of the survey, the participant was redirected to an unlinked information form, where they could provide their personal information to enter an optional draw for a prize.

### 2.5 Data management

In order to protect participants’ confidentiality, participants were asked not to use names during the recorded period of the focus groups. The recording was securely transferred to the youth lead immediately following the consultation and stored on encrypted CAMH computers. No names were attached to the online survey. The survey was hosted on secure CAMH servers.

### 2.6 Support for youth

Youth RAs were trained for this role and continuously supported by CAMH research staff, including remote attendance of the focus groups by a CAMH youth lead. The host organization had mental health support available during the focus groups and immediately after. A debrief after the focus groups addressed any issues or questions that may have arisen for youth RAs during the focus group. Participants were provided with contact information for the youth lead and senior researcher, as well as a list of local mental health resources. Host organizations and participants had the option to receive a copy of the final report of the manuscript and the completed version of the Wellness Quest tool to ensure transparency.

### 2.7 Data analysis

Youth voices guided the analysis, as the data were analysed by a youth lead in consultation with other youth. The youth lead helped prioritize youth needs and relevancy to youth. Focus group interviews were transcribed verbatim, imported into NVivo (12 Pro) and analysed by a youth lead using qualitative thematic analysis and an inductive approach to identify common themes. A youth lead continually read the transcripts to become familiar with the content, enabling insight into initial thoughts and emerging themes within focus groups. The words and sentences that conveyed similar meanings were identified and labelled as codes, allowing segments of text to be interpreted and categorized.^25^ Following coding, categories were developed and discussed to develop broader overarching themes. Regular youth lead debriefs with experienced researchers ensured research methodology was followed and data analysis and codes were objective. A second coder used the codebook to code two transcripts to ensure rigour and to confirm the consistency of the analysis.^26^ Cohen's Kappa of inter‐rater reliability was 0.80. Differences in coding were discussed between the two coders, and agreement was reached in all instances. The results of the survey were summarized using descriptive statistics.

## 3 RESULTS

### 3.1 Participant characteristics

**FIGURE 1:**
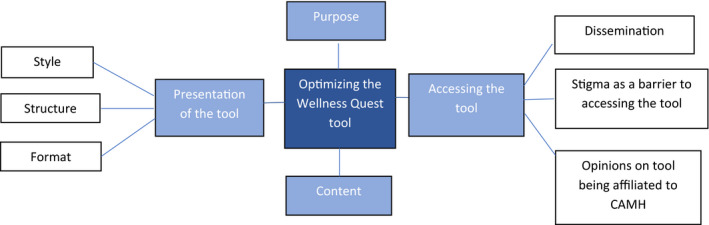
Themes related to the content, presentation, and utility of the Wellness Quest tool

There were 52 focus group participants, aged 14 to 25 (M = 19.85, SD = 2.61; four missing). There were between 8‐14 participants in each of the five focus groups. For the online survey, the 25 participants were aged 17 to 26 (M = 22.36, SD = 2.48). Sixty percent of survey participants came from Ontario, 20% from Quebec, while the rest were from Alberta, British Columbia and Saskatchewan. The sample was largely from urban areas of Canada. A majority of participants were either working, studying or volunteering. Twenty percent of focus group participants and 24% of survey participants classed themselves as being currently unemployed. English was a first language for 67% of participants in the focus group and 84% of participants in the survey. Demographic information is presented in Table 2. Themes related to the content, presentation and utility of the Wellness Quest tool are presented in Figure 1.

**TABLE 2:** Demographics of participants in the focus groups (n = 51) and national survey (n = 25)

Participant characteristics	Focus groups		National survey	
	No	%	No	%
Gender identity				
Female	34	66.7	19	76.0
Male	11	21.6	2	8.0
Transgender or gender diverse	5	11.9	4	16.0
Background				
Caucasian	25	49.0	16	64.0
South Asian	8	15.7	5	20.0
East/Southeast Asian	8	15.7	3	12.0
Black	6	11.8	2	8.0
Indigenous	6	11.8	1	4.0
Current occupational status				
Studying	22	43.1	14	56.0
Working	26	50.9	15	60.0
Volunteering	11	21.6	8	32.0
Unemployed	10	19.5	6	24.0
First language				
English	34	66.7	21	84.0
Previously accessed mental health/addiction services	37	73.0	18	72.0

### 3.2 Purpose of the tool

Participants felt the Wellness Quest tool could help first‐time mental health service users to better understand and access the system.

[Participant 1]: I feel like it's a good starting point if you are new to accessing mental health support. It's a good way for you to start thinking about what's available to you.

Participants believed that the tool would be beneficial at different points in people's lives. Participants also strongly believed that the tool would be able to provide youth the knowledge and resources to advocate for themselves and make educated decisions when it comes to their mental health care.

[Participant 2]: You know, when I think back to when I was receiving care, 90% of the time the healthcare worker is telling you what to do, "Try this." "Do this." "Do that." And very rarely do we ask, "What do you want?" "What do you think?" And I think part of the reason is they think that, well, we're youth, we're young people, what do we know? We're probably uninformed. But if I'd have had this tool, I could prove them wrong and say, "No. I do know a lot and this is what I think I need." And it kind of changes the dynamics of the conversation.

Additionally, participants reported that the tool could be accessed not only by youth seeking care, but by people who support their children, family, and friends with mental health difficulties. Youth also mentioned that the tool would be able to fill in a gap within current mental health services.

[Participant 3]: This tool is not only useful for youth, but also useful for healthcare providers. As opposed to say, you know, ‘just follow up with your family doctor’ or something. It would be a lot more useful.

### 3.3 Content

Positive feedback was provided on the content of the Wellness Quest tool throughout all of the interviews and in the survey data (Table 3). Participants described the tool to be effective in what it set out to achieve: education and self‐advocacy.

[Participant 4]: I think just realising that it has a lot of potential and it's a really good resource for young people to have, especially if they don't know – it can be really overwhelming just having a conversation about mental health for a lot of people, so it's a good place to kind of guide them on where they should go.

**TABLE 3:** Selected responses from national survey

Survey questions	Responses	N	%
I understand how to fill out the Checklist	Yes	22	88
The Checklist has all the aspects of care that are important to me	Yes	20	80
The survey response measures provide enough options for me	Yes	22	91
How would you like to access the tool?	Online tool (website/app)	20	100
	Printable resource	11	44
	Physical booklet	11	44
If it is an online resource, where should it be hosted?	On its own website	18	78
	Mental health agency website	3	13
Where would you like to use the tool for the first time?	On my own	17	68
	With a peer at a service agency	8	36
	With a service provider/family doctor	6	24
What would be the best place to advertise the tool?	Social media	21	84
	My school	18	72
	Mental health provider	17	68
The Checklist would be useful to me	Yes	15	79
The Guide would be useful to me	Yes	16	84
I would recommend this tool to a friend	Yes	17	90

Suggestions were made to incorporate new sections in future versions. The new sections commonly discussed are described below.

#### 3.3.1 Medication

Participants recommended that a section be added about common medications used to treat mental health difficulties. They also recommended noting that medication is not the only treatment option and that those who are on medication might consider combining it with psychotherapy to see better, longer lasting improvements to their mental health.

#### 3.3.2 Self‐help section

Another recommended addition was a section containing information around self‐help tools that youth could use to self‐regulate on a regular basis or in moments in which it is not possible to access services. Common suggestions included coping skills, helplines, reaching out to social support, and suggestions for light‐hearted activities.

[Participant 5]: Creating a self‐care box, a mental wellness box, so these are things that help you cope or help you manage.

#### 3.3.3 Confidentiality and parental involvement

Participants requested information about their rights as youth when accessing a service, as well as confidentiality and situations that lead to a breach of confidentiality. Participants also felt that youth should be informed that helplines are available for those who prefer anonymity when seeking support. Participants recommended that a disclaimer be placed at the beginning of the tool stating that this resource can be accessed discreetly so as to maintain privacy.

[Participant 6]: I think it might be beneficial to brainstorm some of the common fears that people have around confidentiality. You could kind of like answer those questions, like ‘no, this can't be brought up against you if you want to get into a certain programs’, ‘no this can't be checked if you want to get into a certain career’.

[Participant 7]: A few years ago if I had seen this, I would have immediately been like ‘no I don't want to because then my parents are going to know’.

Additional recommendations included a directory of services and a series of real‐life testimonies of youth who had accessed mental health care. Participants also suggested adding a section on signs and symptoms of common mental health disorders. However, some participants were hesitant about this addition, as this information could lead to self‐diagnosis. Additional suggestions for new sections included alternative therapies, different forms of psychotherapy, and sexual assault and harassment services. Information about services for specific populations was also recommended, such as Indigenous youth, immigrants, refugees, youth with different religious backgrounds and 2SLGBTQ+ (Two Spirit, Lesbian, Gay, Bisexual, Trans, Queer and Questioning) youth. They further suggested adding a glossary to describe clinical terms.

### 3.4 Presentation

#### 3.4.1 Style

##### Language and wording

Participants throughout the focus groups and survey stated that the language used in the checklist and guide was inclusive, professional, easy to understand and to the point. Participants also shared positive feedback on the name ‘Wellness Quest’ and recommended adding a subtitle to ensure people are aware that it is a mental health literacy tool. Some participants suggested making the title more personal by naming it ‘Your Wellness Quest’.

[Moderator]: So, it (the title) caught your eye?

[Participant 8]: Yeah. I feel like it does its job, being geared towards the younger people.

[Participant 9]: It sounds positive.

[Participant 10]: Yeah, it's very inclusive.

Proposals were made to re‐word specific phrases that came across as ‘harsh’. For instance, with the phrase ‘someone who can understand *your issues’*, the words ‘your issues’ could be changed to ‘your situation’. This opinion was also expressed in the survey. Participants also believed that some of the language used in the guide was too informal, such as the statement ‘when you feel like crap’. While this language was intentionally used in an attempt to be relatable to youth, participants felt that the language should be kept professional and formal, including using fewer exclamation points.

##### Graphic design

Words like ‘colourful,’ ‘brighter,’ ‘bolder’ and ‘more engaging’ were frequently used to describe what the tool should look like in order to be youth‐friendly and accessible. Some participants recommended using a fictional character to guide the individual through the ‘quest’, especially for younger users of the tool. These suggestions were also supported by survey results. However, it was suggested that there should also be an option of a simpler format of the tool, for older users of the tool or those who prefer this for accommodation and accessibility reasons.

[Participant 11]: I feel everybody I've talked to or come across, myself included, have been very mature people, because we've been forced to grow up and mature quickly dealing with all of this, so this is a fairly mature thing but still aesthetically pleasing. It's not super bright in your face, but it looks nice, so having something like that.

[Participant 4]: I feel like it needs to be like capture more of a happy mood because if you're seeking mental services… it doesn't have depressing pictures and depressing colours all over it.

[Participant 12]: I think maybe even having an option between, like a simple format, whether it's accessibility reasons that you need, you can't deal with all the extra animation and all that, or you're just coming in with a kind of more of that approach, and then having a kind of a more engaging, for the younger audience, or kind of like a less clinical approach that makes it more accessible for people.

#### 3.4.2 Structure

##### Length and structure

Multiple participants described the guide as being too wordy. However, other participants stated that the length was not a problem as all the information was useful and users only had to read sections relevant to them. Structurally, participants felt that the guide would benefit from an index and separate sections to allow readers to access the specific sections they need and avoid feeling overwhelmed by the length of the tool. They also felt that each new section should start with a short description of the content.

##### Scaling of the checklist

The checklist helps young people identify relevant types of services, potential partners in treatment, and accessibility concerns that are most important to them. The checklist asks the question ‘how important is this to me?’ with responses on a 4‐point Likert scale with the response options of ‘very’, ‘a bit’, ‘not’ and ‘not applicable’. Some participants in the focus groups and survey believed the checklist scale should be reworded.

[Participants 13]: I It is still important even if it doesn't apply to you.

The majority of participants in the focus group and the survey were satisfied with the use of this question and scale as they found it straightforward and easy to use.

[Participant 14]: I just want to say, compared to other questionnaires I filled out, this is probably the simplest one, and it was really easy for me to fill out, and very quick. And it wasn't very ambiguous either…

However, a few participants suggested it would be beneficial to use ‘yes/no’ responses.

#### 3.4.3 Format

Participants wanted both a physical copy and online version of the tool. Participants recommended having a physical copy available, especially in more remote areas where not everyone may have access to the internet or in areas/agencies accessible for street‐involved youth. They suggested that the printed tool be shorter and more condensed, such as in a pamphlet format. They suggested that this version include quick and important information, with web‐links and scan codes allowing readers to access more material online.

[Participant 15]: I think it would also be important to have some kind of (…) a paper version, just to make it accessible, whether it's like up in northern Canada that doesn't necessarily have as much access to the internet and stuff.

Participants in both the focus groups and the survey wanted a Wellness Quest website or app. They expressed that an online tool would allow it to be up‐to‐date, user‐friendly, interactive and easily accessible to a wider population. They suggested the digital version should have extensive information separated by sections.

[Participant 16]: I think what would be really helpful, if you have, like, an online version of the guide, because then maybe you could just have a table of contents and you could click where you want to go, instead of looking through the whole thing, or be able to search. I think that would save time and be pretty helpful, especially for youth, because we don't like to do a lot of reading.

Survey and focus group participants suggested online accounts in which users could save their progress and return, or a downloadable PDF version enabling users to highlight text and add personal comments. To promote accessibility, participants suggested a ‘read aloud’ option for the digital version. Moreover, they felt that the digital tool should have options for content to be translated to multiple languages and that the print version could have the title in different languages.

### 3.5 Accessing the tool

Youth in both the qualitative and quantitative components reported that social media advertising could be used to rapidly increase awareness of the tool. Survey results showed that the most popular recommended platforms were Facebook, Instagram, YouTube and Twitter.

Participants suggested that the tool may be able to work hand‐in‐hand with health‐care services, mental health organizations, schools and universities. This would involve professionals recommending the tool to youth, with physical copies of the tool being available at their locations, such as doctors’ offices. Additionally, they suggested that university and health‐related websites could feature the tool.

[Participant 17]: Definitely reach out to universities. Have them put it on their website, and then have the university send it out to all their students, because like students are very keen on, you know, checking e‐mails at least when they're at university. And they also like leveraging organizations (…) If it's online I just can have them like, "Alright everybody, take out your phone, go to this website right now." And let's say it's very easy to type like WellnessQuest.com or WQ.com and it's like right there and it's like, "Okay, now I see it, I typed it" I have like some muscle memory, like I've done this before.

#### 3.5.1 Stigma as a barrier

A few participants mentioned stigma around others seeing them accessing the tool and would prefer to access the tool privately. This opinion was seen in both the focus groups and the survey (see Table 3). However, participants believed that more people should be aware of this tool and in turn, this awareness and education about mental health could help reduce stigma.

[Participant 13]: I know I'm alone in my room and I'm going to lock the door, so nobody walks in, like, while you're on this website. As if I was watching porn, like because it's so stigmatized, right? (…) So I think at the very first time, this is almost like spiritual or very intimate thing (…) like ‘this is my vulnerabilities, this is what I need.’ So I would imagine, at least for me, it would be a very personal thing.

#### 3.5.2 CAMH affiliation

Some participants expressed a positive opinion about a CAMH affiliation on the tool, as this would provide credibility. However, it was stated that the CAMH branding should not be overpowering. Furthermore, participants believed that a CAMH affiliation may encourage health‐care workers to recommend the tool.

## 4 DISCUSSION

This study evaluated young people's perceptions of the Wellness Quest tool. Both qualitative and quantitative findings demonstrate that participants were satisfied with the tool and able to understand the content; they also believed the tool was effective in what it set out to achieve: education and self‐advocacy. They felt that the tool could be used by youth as an educational resource during different times in their mental health journeys, but also by those supporting loved ones with mental health difficulties. Participants stated that they would recommend this tool to their friends.

Suggestions were made to ensure relevance to a wide range of diverse youth with different experiences, including new sections with a diversity focus and multiple language options. Participants agreed the tool should be available in two complementary versions: a comprehensive online platform and a brief hardcopy for those without internet access to ensure equitable access despite the digital divide.^27^ However, participants felt that the current version of the guide was easy to read and provided clear definitions for difficult/clinical terms.

Stigma, consent, privacy and confidentiality were of high importance to participants in the present study when accessing both the tool and mental health services. These findings align with a systematic review of 22 published studies of perceived barriers or facilitators to mental health help‐seeking in youth.^7^ In that review, the prominent barriers included public and self‐stigma related to mental illness and help‐seeking, confidentiality and trust, difficulty identifying the symptoms of mental health, lack of accessibility, self‐reliance, and lack of knowledge about mental health services. If the Wellness Quest resource can be used privately and confidentiality in non‐stigmatizing ways, it may contribute to facilitating the use of mental health services among youth.

Design suggestions included a pleasant aesthetic avoiding ‘typical depressing’ images, with the institutional logo to increase credibility. Similarly, an evaluation of an Australian Mindfulness intervention for youth revealed that youth wanted pictures of young people ‘being happy, being active and having fun'.^28^ Likewise, a study testing a mobile‐based sexual health intervention for young people found that youth wanted colours that match the national health services logo, combined with advertising in reputable local pharmacies and social media platforms to make the resource look credible and ‘more serious’.^29^ This also reflects previous studies on youth, digital media and credibility.^30^, ^31^ It is important that youth resource designers hear the preferences of young people and move towards pleasant, upbeat designs with embedded credibility indicators.

Young people want to be actively involved in the health‐care decision‐making process and report feeling more in control when they are able to voice their opinions and be heard.^32^ However, service users are rarely involved in making decisions when accessing services, and their preferences and goals may be disregarded.^33^, ^34^ Interventions that help youth be actively involved in their care show improved quality of life and satisfaction, which in turn may promote better service engagement.^35^ Improving mental health literacy may improve service utilization and mental health.^8^, ^36^, ^37^ Young people also express the unmet need for developmentally appropriate, relevant and accurate information to enable them to make informed decisions about their mental health.^38^, ^39^ Tools such as the Wellness Quest are specifically designed to promote help‐seeking in youth, not only by increasing awareness of services available to young people, but also by providing information on barriers to help‐seeking. Along with increasing mental health literacy, the Wellness Quest tool may facilitate help‐seeking and equip youth with the knowledge to make informed decisions and advocate for their own mental health.

### 4.1 Youth Engagement

Youth Engagement is increasingly being seen as vital in creating supportive and relevant services for young people and improving the ecological validity of the research.^40^, ^41^, ^42^, ^43^ This project successfully engaged young people at every step, from defining the central need and designing the tool to evaluating the tool. Youth were engaged in a variety of ways, based on their level of interest, availability, commitment and skill.^16^, ^43^ Youth RAs were able to be well trained, identify host agencies within their communities, and support a robust recruitment strategy using their networks; this greatly facilitated recruitment success for a national project. Engaged youth gained knowledge and skills in research, project management, facilitation and tool development. The positive study results may reflect the fact that the tool was designed by youth, for youth, to help to ensure that it is accessible, engaging and non‐stigmatizing for young people.

However, our successes were not without challenges. The project involved a large time commitment for youth. This highlights the importance of compensating youth for their work and ensuring that their engagement is helping them meet their personal goals. The youth RA onboarding process highlighted the lack of standardized research orientation for youth RAs. The training currently available was not the most appropriate or feasible for youth partners in this role. In the future, adaptations are needed to best suit the needs of youth researchers. Finally, a protocol deviation occurred in the age range of youth recruited in the study, affecting one participant. The Research Ethics Board approved the deviation and the team has worked together to ensure that it does not occur in future studies.

### 4.2 Limitations

Some limitations are to be noted. During the focus groups, general discussion about opinions on mental health and services was initiated as an ‘ice breaker’. However, in some instances, these conversations were lengthy. While this may have helped increase rapport, it may have also interfered with discussions of the tool. Additionally, recruitment was dependent on youth RAs' connections and may have been limited to their community network. Furthermore, it was assumed that participants did read over the tool before attending the consultation; however, this was not verified and may have hindered the collection of more detailed feedback.

It should be noted that this study captures a particular section of youth experiences. Participants were mostly English‐speaking, largely resided in urban areas and had internet access. The survey was only accessible online. The majority of both samples was female. Additionally, the study evaluated an English‐only guide, which may have limited the diversity of participants, such as Francophone and newcomer youth. The sample aimed to be diverse; however, some subgroups may have not been represented. Future research should strive to include a more diverse sample of young people and larger sample sizes.

### 4.3 Conclusion

The help‐seeking journey of young people can be overwhelming, non‐linear and often reliant on a young person's ability to find appropriate mental health services. Using a youth‐led design, this study provided insight into the content, design and feasibility of a unique Canadian health literacy and self‐advocacy tool developed by youth, for help‐seeking youth. The Wellness Quest tool may fill a gap within the present mental health‐care system in Canada, by providing youth with developmentally appropriate, relevant information to enable them to advocate for themselves, on their own behalf, and make informed decisions about their mental health care.

## CONFLICTS OF INTEREST

None.

## AUTHOR CONTRIBUTIONS

**Asavari Syan's** contributions include analysis and interpretation of the data, drafting of the manuscript, and manuscript revision. **Janice Y.Y. Lam's** contributions include conception of the design, acquisition of the data, and revisions of the manuscript. **Christal G.L. Huang's** contributions include conception of the design, acquisition of the data, and revisions of the manuscript. **Maverick S.M. Smith's** contributions include conception of the design, acquisition of the data and revisions of the manuscript. **Karleigh Darnay's** contributions include youth engagement coordination and support throughout project and revisions of the manuscript. **Lisa D. Hawke's** contributions include support of the research and data analysis and revisions of the manuscript. **Joanna Henderson's** contributions include support of the research, revisions of the manuscript, and final approval of the version to be published.

## Data Availability

The data that support the findings of this study are available from the corresponding author upon reasonable request and with Research Ethics Board approval.
